# Identifying transglutaminase reaction products via mass spectrometry as exemplified by the MUC2 mucin - Pitfalls and traps^☆^

**DOI:** 10.1016/j.ab.2020.113668

**Published:** 2020-05-15

**Authors:** Liisa Arike, Gunnar C. Hansson, Christian V. Recktenwald

**Affiliations:** Department of Medical Biochemistry, University of Gothenburg, Gothenburg, Sweden

**Keywords:** Mucus, MUC2, Isopeptide bond, Cross-link, Transglutaminase

## Abstract

In order to demonstrate transglutaminase activity in biological samples immunological as well as glutamine- and amine-donor based assays are commonly used. However, the identification of the transglutaminase reaction product, i. e. the isopeptide cross-linked peptides/proteins or the deamidated protein/peptide are often neglected. This article describes a workflow for the detection of the products of transglutaminase-catalyzed reactions. In particular, possible pitfalls and traps that can arise during the mass spectrometry-based identification of isopeptide cross-links are addressed and characterised on actual samples.

Transglutaminases (TGases, E.C. 2.3.2.13) are a family of amino acyl-transferases that can catalyze the Ca^2+^-dependent formation of a covalent bond between the side chains of protein-bound glutamine and lysine accompanied by the release of ammonia. The nature of the enzyme-substrate intermediate complex is a thioester bond, to which nucleophiles like primary amines such as the ε-amino group of lysine or water can perform a nucleophilic attack thereby producing either an isopeptide bond or a glutamic acid residue [1]. TGase activity in cellular extracts is typically detected by the incorporation of either a biotinylated acyl-donor or an acceptor peptide, and subsequent gel electrophoresis and Western blot [2]. Some researchers have demonstrated TGase activity in tissue sections by using an antibody against the TGase reaction product and co-immunostaining against the cross-linked target protein [3]. Despite the usefulness of these approaches they fail to localize the respective glutamine and lysine residue of a TGase-catalyzed isopeptide cross-link. Hence, an essential piece of information is missing since the introduction of an isopeptide bond results in important structural and functional changes of the target protein(s) as it has been shown for the self-multimerization of tissue transglutaminase [4]. In order to map the residues forming the isopeptide bond mass spectrometric (MS) analysis has become the method of choice. Several studies have utilized an *in vitro* approach where synthesized gliadin peptides have been treated with TGase 2 and the cross-linked TG2-gliadin complexes were subsequently identified by MS analyses [[5], [6], [7]]. Other studies for the detection of protein substrates for TGases used a strategy whereby labelled acyl-donor or –acceptor compounds were added to separate reaction mixtures of e.g. a cell or tissue lysate, and the modified residues were later identified by a database search [[8], [9], [10]]. All these approaches have been proven to be very useful for the identification of new putative TGase substrates and the identification of preferred substrate residues. However, they have two major drawbacks. Firstly, it is not possible to identify the “natural” cross-linked protein substrates inside a cell or biological fluid. Second, TGases are promiscuous when it comes to the amine-donor. Therefore, any freely exposed lysine residue or a non-acetylated protein N-terminus of the protein surface can perform the nucleophilic attack and form the covalent bond with the glutamine-donor peptide. Due to the small size of glutamine-donor peptides the influence of steric hindrance when bound to the catalytic cysteine of the enzyme is reduced when compared to a protein substrate, thereby increasing the chance of false-positive identifications.

To overcome these limitations it is necessary to isolate the TGase reaction products from the biological sample before MS analysis. This article describes the workflow for the analysis of cross-linked proteins on the gel-forming mucin MUC2 as an example and points to pitfalls that can arise during this process. Furthermore, possible traps for the analysis of deamidated TGase reaction products are addressed as well.

The gel-forming mucin MUC2 is the major constituent of the intestinal mucus that covers the epithelial surface thereby protecting it from bacterial induced inflammation. MUC2 oligomerises via disulfides involving both the N- and C-terminus. During the later stages of its biosynthesis in the *trans*-Golgi network reduction insensitive bonds between MUC2 monomers are introduced, that have been shown to be isopeptide bonds catalyzed by TGases [11].

One major obstacle for the detection of TGase-mediated cross-linked proteins is that they are far less abundant than linear peptides in a biological sample. Therefore, traditional shotgun proteomics approaches are insufficient as there are too many co-eluting peptides during the LC separation that will suppress the ionization of the cross-linked peptides. To overcome this, it is necessary to enrich for the TGase reaction products via the most suitable method(s), e. g. size exclusion chromatography, SDS-PAGE or affinity purification (Fig. 1A).

**Fig. 1:**
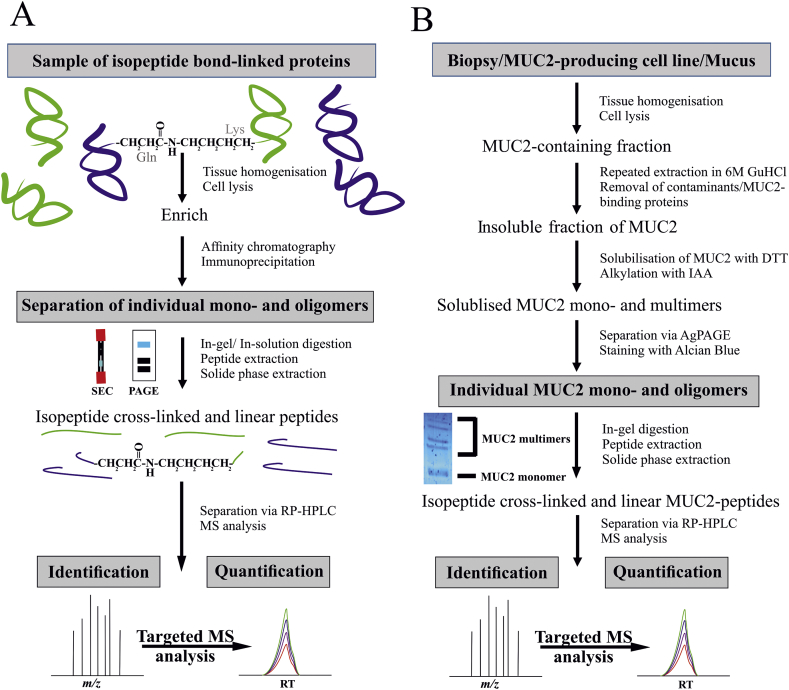
Flowchart of the analytical workflow for the mass spectrometric identification of transglutaminase-catalyzed isopeptide cross-links. Panel A) summarizes a general workflow for the identification of naturally occurring TGase reaction products in a biological sample. Panel B) shows the protocol for the detection of TGase-mediated MUC2-oligomers. The included gel scan shows the composite agarose-PAGE-separated MUC2 monomer and the respective multimers from mouse colonic mucus visualized with Alcian Blue. (For interpretation of the references to colour in this figure legend, the reader is referred to the Web version of this article.)

In the case of the MUC2 mucin, it is possible to take advantage of its insolubility in 6 M guanidinium chloride (GuHCl) and its molecular weight (Fig. 1B). By using repeated extraction steps in GuHCl most other proteins will be washed away. After this MUC2 can be brought into solution by the addition of dithiothreitol (DTT) and it is subsequently alkylated with iodoacetamide (IAA). The molecular mass of MUC2 is 650 kDa for the primary translation product. After extensive *N*- and *O*-glycosylation the mass of the MUC2 monomer increases to approximately 2.5 MDa. Therefore, it is possible to obtain almost pure TGase–based oligomers after separation by composite agarose-polyacrylamide gel electrophoresis (AgPAGE) [12]. After staining of the MUC2 monomer and its multimers with Alcian Blue the bands in the gel are subjected to protease digestion before MS analysis.

As the obtained peptides can become large the choice of the protease can be critical for the outcome of the experiment. This is especially important for the analysis of cross-links where lysine residues are part of the covalent bridge as in TGase-catalyzed isopeptide bonds because the commonly used protease trypsin will not cleave once the lysine residue has been modified [13]. Therefore, the addition of a second protease or choosing a protease with different specificity is beneficial. Chymotrypsin generally produces shorter peptides than trypsin. However, for MS analyses chymotrypsin should be avoided because the new C-termini are aromatic amino acids or leucine which do not easily take up protons, resulting in the suppression of the ionization efficiency. For the digestion of the MUC2 mucin a combination of trypsin and AspN is recommended. The protein bands of the composite gel are first digested with trypsin followed by AspN after blocking the trypsin activity with phenylmethanesulfonyl fluoride. Afterwards, the samples are cleaned from salts and buffer compounds by C18 solid phase extraction and separated on RP-nLC before MS analysis. Since the sample complexity increases by n^2^ for cross-linked peptides high resolving power and mass accuracy become very important for the correct identification of deamidated and transamidated TGase-catalyzed reaction products. We suggest a resolution of at least 60,000 for the parent ions and 15,000 at the MS2 level. During the data analysis the number of false-positive identifications should be limited by setting the mass accuracy to 1–2 ppm for the parent ions and to 10–20 ppm for fragment ions.

There are several software packages available for the analysis of cross-linked peptides e.g. Kojak, pLink, ProteinProspector, StavroX [[14], [15], [16], [17]]. In order to detect cross-linked peptides with these softwares the user has to define its database, the proteolysis of the proteins, static and variable modifications, error tolerances for MS1 and MS2 level as for regular MS database search. In addition, the cross-linked amino acid residues, i. e. Gln and Lys, protein N-terminus for TGase reaction products and the introduced composition/mass (shift) of the cross-linker, i. e. –NH_3_/-17.03 for TGase-catalyzed isopeptide-bonded dipeptides, have to be specified for the detection of the cross-linked products. Therefore, the identification of the TGase-mediated isopeptide cross-link relies on the alteration of the dipeptide mass by the loss of ammonia. Since a cross-link that occurs between two peptides (type 2, inter-link) contains one additional N- and C-terminus in comparison to the same peptide sequence as a linear peptide the net mass gain is one mass unit for TGase reaction products. This raises specific problems for the identification of TGase-mediated cross-links in consecutive peptide sequences containing a protease cleavage site. The deamidated form of this peptide with a missed cleavage will have the same mass as the cleaved and transamidated dipeptide of the same sequence. In fact, they are isomers as the molecule composition changes by -N-H+O for both modifications and therefore have the same parent ion mass. In order to verify if the consecutive peptide sequence has been modified by an isopeptide bond or by a deamidation a regular database search with deamidation of glutamine/asparagine should be performed in parallel. If the same parent ion with an assigned deamidation is detected in this search the two MS2 spectra should be revisited and compared for indicator ions. However, in the absence of indicative ions for one or the other modification it will not be possible to correctly annotate the modification. Fig. 2A and B show an example of such a case from the analysis of mouse Muc2 oligomers where the same precursor was identified as cross-linked and deamidated peptide. Due to the possible annotation of the peaks at *m*/*z* 1258, 1357 and 1428 as y10, y11 and y12 fragments of the deamidated peptide sequence this modification could be assigned (Fig. 2B). On the other hand, the same spectrum contains the b2 to b4 fragments of the α-peptide for the isopeptide cross-linked dipeptide what could argue for this modification (Fig. 2A). Hence, it is not possible to determine which of the two versions is the correct one. This result might be explained by the known observation that TGase-mediated transamidation and deamidation can happen in parallel [18]. It is expected that a linear deamidated peptide with missed cleavage site and the transamidated dipeptide version would elute at different retention times. However, in this particular case the spectra for both products were identified at the same retention time resulting in a mixed MS2 spectrum from two isomeric precursors which prevents a distinct decision for one or the other. This could suggest that both modifications were obtained by TGase activity and exist in parallel. Therefore, indications for the cross-linked product should be taken from orthogonal data like identification of the cross-linked peptide in the dimeric form of the protein, e. g. after an initial separation on a polyacrylamide gel.

**Fig. 2:**
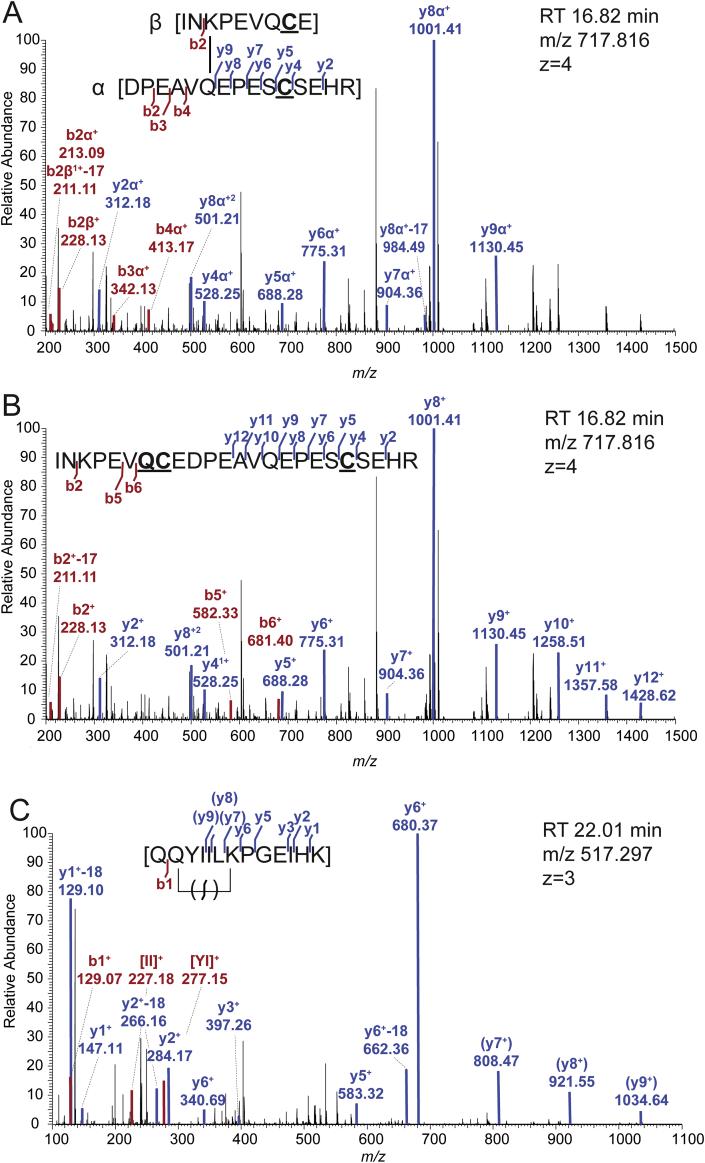
Examples of identified isopeptide cross-links from an MUC2 oligomer. A) MS2 fragment spectrum of the parent ion [M+4H]^4+^ 717.81, annotated as isopeptide cross-link after analysis by the StavroX software and manually inserted into the raw spectrum. B) MS2 fragment spectrum of the same parent ion [M+4H]^4+^ 717.81, annotated as deamidated version using the ProteinProspector search engine. Both annotations cover the same consecutive sequence INKPEVQCEDPEAVQEPESCSEHR corresponding to amino acid residues 197–220 from murine -Muc2. The deamidated version in B) contains a missed cleavage site for AspN N-terminal of aspartate 206. C) MS2 fragment spectrum of the parent ion [M+3H]^3+^ 517.30. Detection of the b1 ion excludes the formation of pyro-glutamate at the N-terminal glutamine. Fragment ions labelled in brackets (y7 to y9) suggest a possible break of the isopeptide bond in the mass spectrometer. [II] and [YI] mark internal fragment ions. Underlined cysteines correspond to carbamidomethylation of the residue, underlined glutamine corresponds to a deamidation.

Another modification that can lead to false-positive identifications of the transamidated reaction product is the formation of pyro-glutamate (pyroGlu). This post-translational modification occurs during LC-MS analysis if the N-terminus of a peptide consists of glutamine or glutamate [19]. For glutamine this results in the same loss of ammonia as for the TGase-catalyzed intra-molecular cross-link in this peptide (type 1, loop). Hence, inspection of the fragment spectrum is necessary to verify the TGase-catalyzed cross-link. Fig. 2C shows an example of such a peptide where pyroGlu formation can be excluded due to the presence of the b1-ion. In this spectrum the presence of the y7 to y9 ions suggest that the isopeptide bond has been broken in the mass spectrometer as has been shown by the work of Kang and Baker [20]. Since this is not a well-studied fragmentation event the respective fragments are labelled in brackets. However, in spectra where pyroGlu formation cannot be excluded the possibility of a cross-link should be discarded.

Finally, in order to quantify and validate the data we suggest a targeted MS method, parallel reaction monitoring (PRM) together with the open source software package Skyline. The PRM method does not require any previous knowledge of fragment ions, therefore it is easy to apply and it improves the spectral quality for low abundant and complex cross-linked peptides. Skyline [21] adds automation of the quantification into the pipeline, which is beneficial when larger datasets have to be analysed. The combination of PRM analysis and Skyline has been applied for chemically cross-linked peptides and protocols for such workflow exist [22,23]. As Skyline is not designed to analyse naturally occurring cross-linked peptides two different approaches can be used depending on which kind of cross-linked peptides are analysed. For TGase-mediated isopeptide bonds in the same peptide, type 1, loop cross-link, loss of NH_2_ on glutamine and loss of H on lysine are sufficient in order to insert the crosslinked peptide into Skyline. For type 2, inter-linked peptides, the identified cross-link needs to be added as a small molecule and the fragment ions will be inserted manually, based on the previous knowledge from the search engine.

In comparison to the detection of TGase-catalyzed transamidated peptides the identification of deamidated reaction products is more straightforward as most search engines provide this post-translational modification as variable modification. However, the identification of deamidated TGase reaction products has also several problems. Therefore, care has to be taken during data acquisition and peak picking. The mass of the first ^13^C peak and the deamidation product differ only by 19 m mass units for charge state one. This can cause wrong assignments of overlapping peaks from the amidated form of the peptide [24]. Hence, the distinction between the two peaks requires high resolving power and the mass spectrometer should be set to a resolving power of at least 60,000. In addition, deamidation can occur spontaneously [25], requiring appropriate controls to be analysed in parallel to the actual sample. This can easiest be achieved by treatment of a sample aliquot with a TGase inhibitor.

In conclusion, the advances in high resolution accurate mass spectrometry make it possible to identify and map glutamine- and lysine-residues involved in TGase-catalyzed transamidation and deamidation reactions. This information on the localization of these sites can provide important information about structural and functional changes in the TGase substrate proteins. For example, identification of the isopeptide cross-links occurring *in vivo* will be useful to answer questions of homo- or hetero-oligomerisation in target proteins and the composition of protein networks. However, the analysis of isopeptide cross-links from biological sources remains challenging due to their low abundance and the potential of false-positive results due to ammonia loss during TGase-mediated cross-linking. Therefore, careful evaluation and appropriate controls are needed in order to verify the obtained results.
